# Semi‐supervised classification of fundus images combined with CNN and GCN

**DOI:** 10.1002/acm2.13746

**Published:** 2022-08-10

**Authors:** Sixu Duan, Pu Huang, Min Chen, Ting Wang, Xiaolei Sun, Meirong Chen, Xueyuan Dong, Zekun Jiang, Dengwang Li

**Affiliations:** ^1^ Shandong Key Laboratory of Medical Physics and Image Processing Shandong Provincial Engineering and Technical Center of Light Manipulation School of Physics and Electronics Shandong Normal University Jinan China; ^2^ The Second Hospital of Shandong University Shandong University Jinan China; ^3^ Department of Medicine The Second Hospital of Shandong University Jinan China; ^4^ Eye Hospital of Shandong First Medical University (Shandong Eye Hospital) Jinan China; ^5^ State Key Laboratory Cultivation Base Shandong Provincial Key Laboratory of Ophthalmology Shandong Eye Institute Shandong First Medical University and Shandong Academy of Medical Sciences Jinan China; ^6^ School of Ophthalmology Shandong First Medical University Jinan China; ^7^ Affiliated Hospital of Shandong University of Traditional Chinese Medicine Jinan China

**Keywords:** attention mechanism, diabetic retinopathy, graph convolutional network, semi‐supervised classification

## Abstract

**Purpose:**

Diabetic retinopathy (DR) is one of the most serious complications of diabetes, which is a kind of fundus lesion with specific changes. Early diagnosis of DR can effectively reduce the visual damage caused by DR. Due to the variety and different morphology of DR lesions, automatic classification of fundus images in mass screening can greatly save clinicians' diagnosis time. To alleviate these problems, in this paper, we propose a novel framework—graph attentional convolutional neural network (GACNN).

**Methods and Materials:**

The network consists of convolutional neural network (CNN) and graph convolutional network (GCN). The global and spatial features of fundus images are extracted by using CNN and GCN, and attention mechanism is introduced to enhance the adaptability of GCN to topology map. We adopt semi‐supervised method for classification, which greatly improves the generalization ability of the network.

**Results:**

In order to verify the effectiveness of the network, we conducted comparative experiments and ablation experiments. We use confusion matrix, precision, recall, kappa score, and accuracy as evaluation indexes. With the increase of the labeling rates, the classification accuracy is higher. Particularly, when the labeling rate is set to 100%, the classification accuracy of GACNN reaches 93.35%. Compared with DenseNet121, the accuracy rate is improved by 6.24%.

**Conclusions:**

Semi‐supervised classification based on attention mechanism can effectively improve the classification performance of the model, and attain preferable results in classification indexes such as accuracy and recall. GACNN provides a feasible classification scheme for fundus images, which effectively reduces the screening human resources.

## 1 INTRODUCTION

Diabetic retinopathy (DR) is a microvascular disease that occurs in the retina in diabetes. It is mainly pathologically characterized by retinal vascular changes. The fundus is mostly manifested as retinal exudation and edema, neovascularization, hemorrhage, and the formation of proliferating membrane.

**FIGURE 1:**
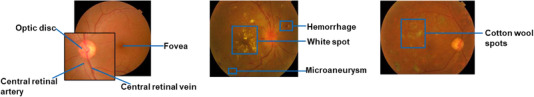
Comparison of normal retina and diabetic retinopathy images

Normal fundus images mainly contain arteries, veins, macula, optic disc, and other structures, while common abnormal lesions in DR images include microaneurysm, hemorrhagic spots, white spots, cotton wool spots, neovascularization, and so forth. Microaneurysm showed red round spots on fundus images, reflecting the changes of vascular performance in the early stage. Hemorrhagic spots are caused by blood leakage from blood vessels to the retina, which presents dark red spots or massive spots on fundus images. The formation of white spots is related to the accumulation of fatty tissue caused by retinal neuropathy. Leukoplakia are bright white plaques caused by nutrients such as lipids and proteins leaking from blood vessels into the retina. The formation of cotton wool spots is related to focal ischemia and necrosis of nerve tissue. Neovascularization is ischemia caused by vascular obstruction, which causes the retina to generate small and disordered neovascularization. The normal fundus images and DR fundus images are shown in Figure 1.

Clinically, according to the presence or absence of retinal neovascularization, DR is divided into non‐proliferative diabetic retinopathy (NPDR) (or simple or background type) and proliferative diabetic retinopathy (PDR).^1^ NPDR is divided into three stages: (1) Stage I: The appearance of microvascular tumor in the fundus. (2) Stage II: White spots with clear edge and irregular shape appeared in the fundus. (3) Stage III: Cotton flocculent leukoplakia appears in the fundus.

**FIGURE 2:**
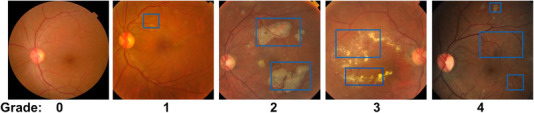
Diabetic retinopathy images severity grading

As shown in the Figure 2, the DR images were divided into five grades according to the severity: normal, mild NPDR, moderate NPDR, severe NPDR, and PDR.

Due to the complex lesions of DR images and different pathological manifestations, the identification efficiency of doctors alone is extremely low and consumes a lot of medical resources. The purpose of this study is to achieve DR images classification by extracting pathological features from DR images.

In recent years, deep learning is undergoing rapid changes, and has achieved remarkable achievements in image processing, semantic recognition. Nayak et al.^2^ proposed to classify the fundus images into three categories: normal, proliferative, and non‐proliferative by using exudation and vascular regional texture combined with neural network; Adarsh et al.^3^ used image processing technology to identify blood vessel, exudate, microaneurysm, and other lesions with texture features in retinal images, so as to classify DR lesions; Prentasic^4^ used convolutional neural network (CNN) to detect exudates in color fundus images, which improved the performance of DR diagnosis. Liu et al.^5^ proposed to classify optical coherence tomography (OCT) images by extracting texture features.

Li et al.^6^ trained the visual geometry group 16 (VGG‐16) model with deep transfer learning. They detected and classified age‐related macular degeneration (AMD) and diabetic macular edema (DME) images through OCT images, and showed better performance in retinal image classification. Schlegl et al.^7^ used a CNN framework based on encoder–decoder to detect intraretinal cystoid fluid (IRC) and subretinal fluid (SRF), which achieved a classification accuracy of 0.94 for OCT images. Christopher et al.^8^ evaluated and compared the performance of VGG‐16, Inception‐v3, and ResNet models. Transfer learning method is used to improve the detection performance of glaucomatous optic neuropathy (GON) and accelerate the convergence of the model.

In order to better implement the deep learning mechanism, Shanthi et al.^9^ proposed an improved AlexNet architecture for DR classification, which improved the classification performance. Shankar et al.^10^ proposed a new automated hyperparameter tuning perception‐v4 (HPTI‐v4) model for DR classification of color fundus images.

Deep neural network has a good effect on feature extraction ability. In addition, due to the interactive relationship between different lesion types of retinopathy, we use graph convolutional network (GCN) to describe this relationship, and combine it with the image features extracted by CNN to classify the images.

The concept of graph neural network (GNN) was first proposed in 2005. In 2009, Franco^11^ defined the theoretical basis of GNNs in his paper. GCN is the most commonly used network, which belongs to a branch of GNN. In 2013, on the basis of graph signal processing, Bruna^12^ first proposed a CNN based on the frequency domain and based on the spatial domain on the graph. In fact, graph convolution based on frequency domain can be regarded as a special spatial method.

GCN has been widely used in small sample learning,^13^ point clouds,^14^ image classification,^15^ and other tasks, and achieved good results. Ma et al.^16^ designed a pooling operator based on Fourier transform and combined it with GCN for graph classification, achieving good performance on multiple datasets. Lin et al.^17^ used a method based on GCN and self‐supervised learning to classify fundus images.

In order to enhance the feature extraction capability of the network, attention mechanism is introduced in many tasks, which can make the network to focus on important information and reduce information loss. Bahdanau et al.^18^ applied the attention mechanism to the field of NLP for the first time. Petar et al.^19^ proposed graph attention networks (GAT), which applied the attention mechanism to GCNs, which effectively improved the generalization ability of the model.

Semi‐supervised learning is a hot topic in the field of medical image research. It combines labeled data and unlabeled data for learning. The basic idea is to optimize the model established by labeled data by using unlabeled data. The common semi‐supervised learning methods include graph‐based semi‐supervised method, co‐training method, generative method, and so on. Qiao et al.^20^ combined co‐training with deep network, regarded multiple subnetworks as multi‐view networks, and used adversative samples to improve view diversity. Ghorbani^21^ used a semi‐supervised approach to adjust the loss function, and proposed an end‐to‐end learning method for semi‐supervised classification. Sohn et al.^22^ designed a semi‐supervised learning framework combining self‐training and consistency regularization for target detection. The teacher model is trained to generate pseudo labels of unlabeled images, and the false labels are enhanced to update the model.

On the basis of previous work, we propose a new model, which combines CNN and GCN to enhance the ability to capture image information, and uses attention mechanism in the graph convolution module to reduce information loss.

Our main contributions can be summarized as follows:

1. In order to improve classification accuracy, our network use both CNN and GCN to learn the features and node relationships of multi‐label fundus images. The network uses max‐pooling layer to make the image retain more detailed information.
2. The graph convolution module based on the attention mechanism can effectively capture important node information of fundus images, extract structural features, and construct node sequences.
3. We use semi‐supervised learning to improve the learning performance, solve the problem of weak generalization and inaccuracy of the model, and improve the classification performance of fundus images.

## 2 METHODS

### 2.1 Overview

**FIGURE 3:**
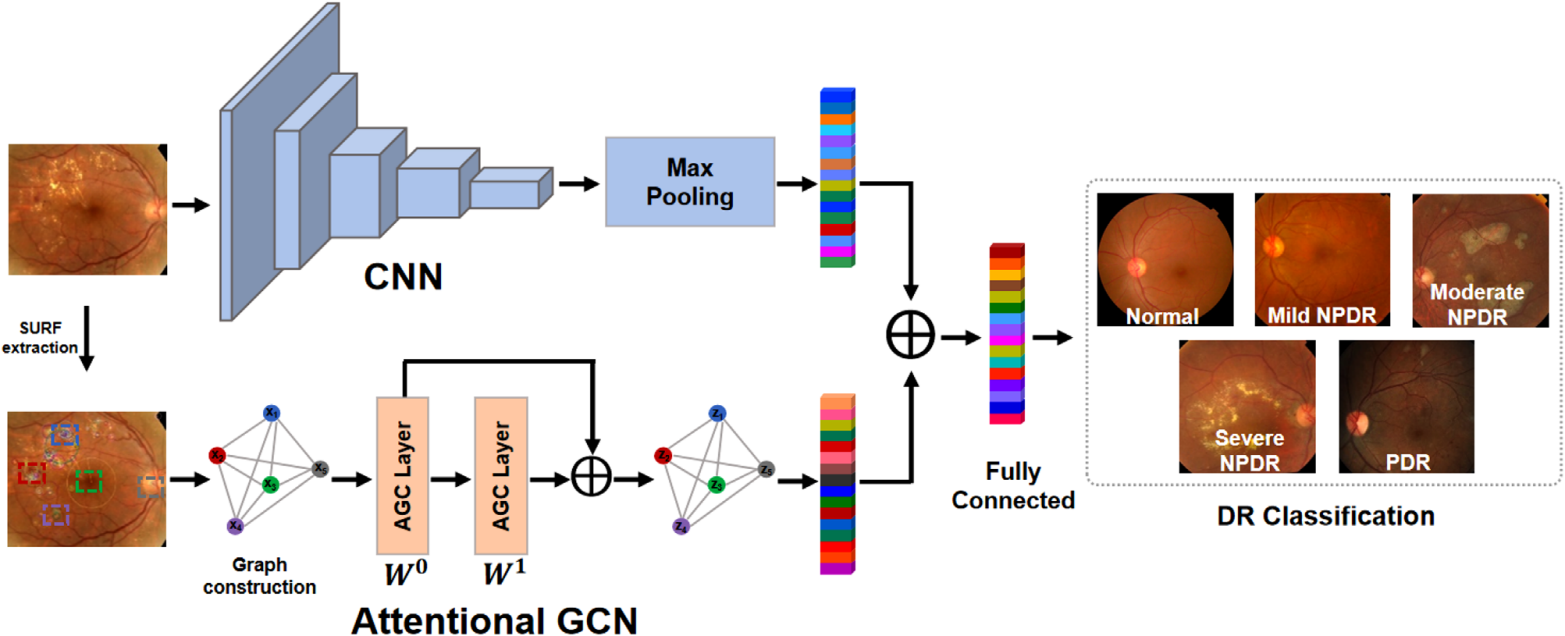
Architecture of the proposed network for classification of fundus images

This section describes our network in detail. The network is constructed based on the improved VGG‐19^23^ and GCN.^24^ The network structure is illustrated in Figure 3, which consists of three parts: the CNN module, the GCN module based on the attention mechanism, and the classifier. In the CNN branch, we use a modified VGG‐19 network to extract image features and output a feature vector. For the GCN module, the attention mechanism is introduced to generate valuable feature from every single hop of the graph convolutional layer to reduce noise and redundancy from input and capture node features. The attention mechanism can help us solve the problem of selecting the order of graph nodes. The attention graph convolutional (AGC) module uses the attention mechanism to replace the fixed standardized operations in the graph convolution. Finally, the information captured by the two parts is fused and input into the classifier for classification.

### 2.2 CNN module

In Figure 3, our CNN module is a refinement of VGG‐19. As shown in Table 1, for VGG‐19, we delete the max‐pooling layer in the last convolutional block and keep other max‐pooling layers and all convolutional layers in the model. Finally, we replace the following three fully connected layers with a max‐pooling layer.^25^ The output of the last convolutional layer of the CNN module is a three‐dimensional feature map with the size of 14 × 14 × 512, which is transformed into a feature vector with the length of 512 by max‐pooling layer. The max‐pooling layer retains the maximum value of the region and preserves the features with high recognition, which can reduce the migration error caused by the convolutional layer parameters. Max‐pooling can ensure the position and rotation invariance of features and retain more texture features of images. In addition, it reduces the number of parameters and can compress the size of model well.

**TABLE 1:** Architecture of VGG‐19 network

**Layer**	**Patch size/stride**	**Output size**
Conv×2	3 × 3/1	224 × 224 × 64
Pool	2 × 2/2	112 × 112 × 64
Conv×2	3 × 3/1	112 × 112 × 128
Pool	2 × 2/2	56 × 56 × 128
Conv×4	3 × 3/1	56 × 56 × 256
Pool	2 × 2/2	28 × 28 × 256
Conv×4	3 × 3/1	28 × 28 × 512
Pool	2 × 2/2	14 × 14 × 512
Conv×4	3 × 3/1	14 × 14 × 512
Max‐pooling Layer	14 × 14/1	1 × 1 × 512

### 2.3 GCN module

#### 2.3.1 Graph construction

We employ graph to describe the correlation between various lesions, and use speeded up robust features (SURF) algorithm^26^ to detect the possible lesion regions of DR images. Figure 3 shows the results of feature points' detection of fundus images using SURF algorithm. The detected objects are represented as graph nodes, each of which is connected to all other nodes except itself.

Suppose $y_{i}$ represents the spatial center coordinate vector of node *i*. The region information of each node is defined as$\;\{m_{i},n_{i},w_{i},h_{i}\}$, where $m_{i}$ and $n_{i}$ are the upper‐left coordinates of the node, $w_{i}$ and $h_{i}$ are, respectively, the width and height of this region. Node *i* can be denoted as $y_{i}=\;\left(m_{i}+\frac{w_{i}}{2},n_{i}+\frac{h_{i}}{2}\right)$. The connection weight between nodes *i* and *j* is calculated as follows:

(1) $$w_{ij}=\left\{\begin{array}{cc} e^{-\frac{{\left\|y_{i}-y_{j}\right\|}_{2}^{2}}{2\omega^{2}}}, & \mathrm{if}\;i\in N_{k}\left(j\right)\;and\;j\in N_{k}\left(i\right)\\ 0 & ,\mathrm{otherwise} \end{array}\right.$$

where ω is the parameter and is set to 1.6. $N_{k}\left(j\right)$ represents the set of k‐nearest neighbor (KNN)^27^ of vertex *i*. We use Euclidean distance to measure the distance between two nodes and set *K* is 8. When both node *i* and *j* are within each other's KNN range, it is judged that there is a connection between the two nodes.

#### 2.3.2 Graph convolutional network

Graph in GCN refers to the topological graph in which vertices and edges are used to establish corresponding relationships in exponential science. GCN directly operates on a graph, and outputs the embedding vector of nodes according to the nature of the neighborhood of the node.^28^ Define a graph as G=(V,E), where $V=\{V_{i}|i=1,\text{…},n\}$ is the vertex set of the graph, and *E* is the linkages between nodes. $X=\left[x_{1},x_{2},\text{…},x_{n}\right]\in R^{n\times m}$ is defined as the feature matrix, where *n* is the number of nodes, *m* denotes the feature dimension, and $x_{i}\;$is the feature vector of the node *v*. $A\in R^{n\times n\;}$ is the adjacency matrix of graph *G*, which represents the adjacency relationship between any two vertices, adjacency is 1, and non‐adjacency is 0. *D* is the degree matrix and $\;D_{ii}=\sum_{j}A_{ij}\;$. The element on the diagonal is the degree of the vertex, that is, the number of elements linked by the element. The output features of a single layer GCN are calculated as follows:

(2) $$H^{\left(1\right)}=\;f\left(D^{-\frac{1}{2}}AD^{-\frac{1}{2}}XW^{0}\right)$$

where $H^{\left(1\right)}\in R^{n\times k}$ is the *k*‐dimensional node feature matrix and $H^{\left(0\right)}=\;X$. $W^{0}\in R^{m\times k}$is a weight matrix. f(x) is an activation function, such as ReLU(x)=max(0,x). According to the above‐mentioned one‐layer convolution formula, the calculation formula for the multi‐layer graph convolution with depth *j* is as follows:

(3) $$H^{\left(j+1\right)}=\;f\left(D^{-\frac{1}{2}}AD^{-\frac{1}{2}}H^{j}\;W^{j}\right)$$

#### 2.3.3 Attention module

**FIGURE 4:**
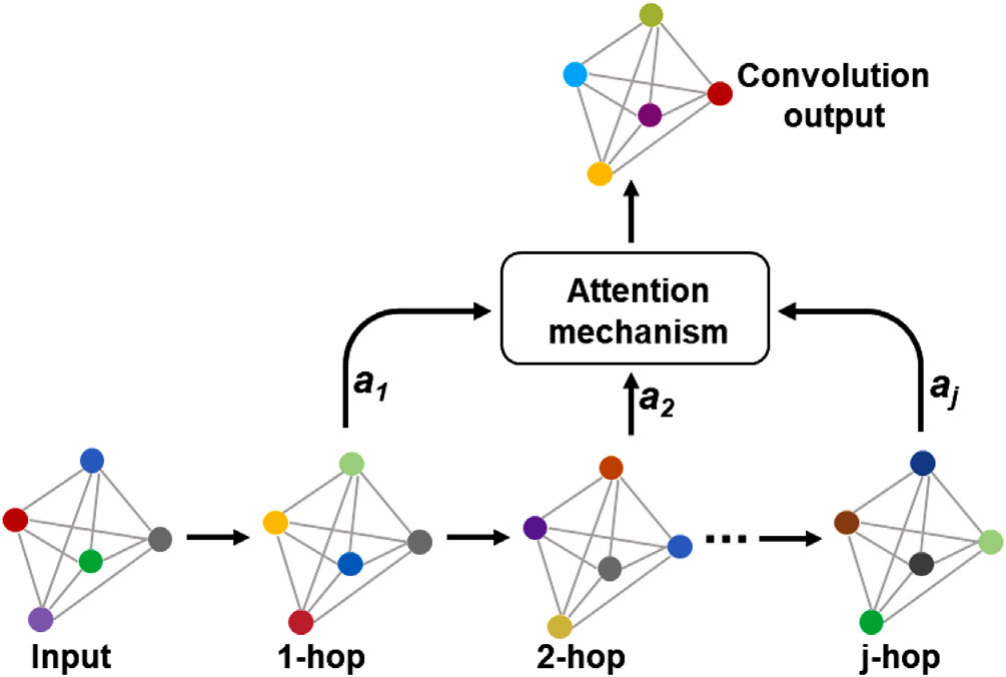
Attention graph convolutional layer

In the graph convolutional layer, a convolutional operation is performed on the neighbors of each node, and the node is updated with the result of the convolution. Then through the activation function and a convolutional layer, until the number of layers reaches the desired depth. In this process, the convolutional results of each layer can only be used for the next convolutional operation, resulting in a large amount of information loss.^29^ In order to reduce the loss of information and suppress useless information, we use the attention mechanism to extract important information in each convolutional operation and construct the AGC layer as shown in Figure 4.

Due to different structure information of different volume layers, we use attention mechanism to aggregate important information in each convolutional step, and get the combination of nodes for each layer as follows:

(4) $$\gamma_{v}=\sum_{i=1}^{j}a_{i}H_{v}^{j}$$

where $a_{i}$ is the attention weight of each hop, and $H_{v}^{j}$ represents the structural feature of node *v* in *j* hop. Graph *G* can be represented by nodes as:

(5) $$G=(\gamma_{1}\gamma_{2},\text{…},\gamma_{v})$$

### 2.4 Loss function

Our model adopts semi‐supervised approach for classification.^30^ First, the labeled samples are used to calculate the parameters, and then the label of unlabeled nodes is obtained by forward propagation.

We suppose that $f_{k}$ represents the features extracted by the CNN module and $h_{k}$ represents the information extracted by GCN module. The image features extracted by the two branch modules are combined as follows:

(6) $$z_{k}=\;\rho \left(x\right)\cdot \left(f_{k}⊕\mu h_{k}\right)$$

where ρ(x) represents the weight matrix of the fully connection layer, and μ is used to adjust the fusion ratio of the features of the two modules, ⊕ represents element‐wise addition. In our experiment, the value of μ is 1.

The train set is randomly divided into labeled data and unlabeled data, and both kinds of data are used for training. In the training process, after every weight update, the prediction of unlabeled data is regarded as pseudo labels. We denote the labeled dataset as *S* and the unlabeled dataset as *U*. For labeled image $S_{i}\in S$, whose corresponding ground truth label is $P_{i}$. For unlabeled image $U_{j}\in U$, whose corresponding pseudo label is ${\hat{P}}_{j}$. *Z* represents the final prediction label. We use the cross‐entropy loss function in the training process, and weigh the loss function of labeled samples and unlabeled samples to optimize the model.

For labeled data:

(7) $$L_{\mathrm{label}}=-\underset{k=0}{\sum^{K}}\sum_{S_{i}\in S}\left[P_{i}logZ_{i}+\left(1-P_{i}\right)log\left(1-Z_{i}\right)\right]$$

where *K* represents the number of categories.

For unlabeled sample, the loss function is as follows:

(8) $$L_{\mathrm{unl}}=-\underset{k=0}{\sum^{K}}\sum_{U_{j}\in U}\left[{\hat{P}}_{j}logZ_{j}+\left(1-{\hat{P}}_{j}\right)log\left(1-Z_{j}\right)\right]$$

Thus, the total loss function of our model is:

(9) $$L=L_{\mathrm{label}}+\lambda L_{\mathrm{unl}}$$

where λ is the hyperparameter used to adjust the two terms, and its value is set to 0.1 in this experiment.

## 3 RESULTS

### 3.1 Dataset

Our dataset comes from the public dataset of the APTOS 2019 Blindness Detection challenge, which contains train images and test images. The dataset size is 9.51G and contains 5590 images, of which 3662 are used for training and 1928 for testing. It also contains train.csv and test.csv, which respectively contain the label of each picture in the train dataset and test dataset. The aim of this competition is to analyze the severity of DR, the normal is 0, the worst is 4. The purpose of this study is to use artificial intelligence methods to diagnose DR as soon as possible and avoid the deterioration of the disease.

There are several obvious problems with the data, for example, the image size is not consistent, the image brightness is different, the size, color, and brightness are also different. Training the original image directly increases the difficulty of training, and it is not easy to find the features of lesions. We need to preprocess the images.

There are black areas on the edges of the original fundus image. The black area is meaningless for classification, so the part with lower pixel value of image edge is removed. Take the diameter of fundus image as the side length and the eye center as the image center to cut the image. In the original image, the black areas only exist on the left and right sides of the image, so it only needs to be cropped in the vertical direction. The image size is adjusted to 224 × 224. For the image with color deviation, supersaturation or undersaturation, the ImageEnhance module of PIL tool is used to perform brightness equalization, color balance adjustment, and contrast adjustment, so that different image display effects are more consistent and highlight features. After that, we remove Gaussian blur^31^ from the image to obtain the difference to enhance the image.

As shown in Table 2, the distribution *n* of all types of samples in the dataset is unbalanced. We adopt data augmentation methods such as flipping, rotation, and shift to increase the number of samples of each class to 1800 to balance the distribution of the categories.

**TABLE 2:** Sample distribution of the dataset

**Class**	**DR classification**	**Number of original images**	**Percentage (%)**	**Number of images after enhancement**
0	Normal	1805	49.29	1800
1	Mild NPDR	370	10.10	1800
2	Moderate NPDR	999	27.28	1800
3	Severe NPDR	193	5.27	1800
4	PDR	295	8.06	1800

### 3.2 Evaluation measures

The evaluation indexes of the experiments included confusion matrix, precision, recall, kappa score, and accuracy. The accuracy represents the proportion of correctly classified samples among all samples, and can be expressed as:

(10) $$\mathrm{Accuracy}\;=\frac{\mathrm{TP}+\mathrm{TN}}{\mathrm{TP}+\mathrm{TN}+\mathrm{FP}+\mathrm{FN}}$$

where TP represents the true positive, TN represents the true negative, FP represents the false positive, and FN represents the false negative.

The precision represents the proportion of the number of correctly predicted images in the total number of positive predicted images, while recall determines the number of positive predicted images in all labeled images. The precision, recall, and kappa score can be expressed as:

(11) $$\mathrm{Precision}\;=\frac{\mathrm{TP}}{\mathrm{TP}+\mathrm{FP}}$$

(12) $$\mathrm{Recall}\;=\frac{\mathrm{TP}}{\mathrm{TP}+\mathrm{FN}}$$

(13) $$Kappa=\frac{p_{0}-p_{e}}{1-p_{e}}$$

where *p*_0_ is accuracy. Suppose that the true sample numbers of each class are $a_{1},a_{2},\text{…},a_{K},$ while the predicted sample numbers of each class are, respectively, $b_{1},b_{2},\text{…},b_{K}$, and the total number of samples is *n*. Then $p_{e}\;$is stated as:

(14) $$p_{e}=\frac{a_{1}\times b_{1}+a_{2}\times b_{2}+\cdots +a_{K}\times b_{K}}{n\times n}$$

### 3.3 Methods for comparison

We selected several advanced models for comparison. To ensure the diversity of methods, we selected the following networks: DenseNet121,^32^ DeepWalk,^33^ dual attention graph convolutional network (DAGCN),^29^ DGCN,^34^ Graph REsidual rE‐ranking Network (GREEN),^35^ hybrid graph convolutional network (HGCN).^36^

- **DenseNet121**. This network creatively proposes the dense block, and adds convolutional layer and pooling layer between each Dense Block. In each dense block, any two layers are directly connected. Thus, the input of each layer of the network is the union of the output of all the previous layers, and the feature map learned by this layer will be directly transmitted to all the subsequent layers as input. DenseNet121 greatly reduces the amount of parameters and the reuse of features.
- **DeepWalk**. DeepWalk uses the random walk, which mainly includes random walk and generation of representation vectors. First, the vertex vector representation is extracted from the graph by random walk algorithm, which is regarded as words in the language model, and the sequence of nodes is simulated as sentences in the language. This method applies unsupervised presentation learning to graphs, and can create meaningful representations for large‐scale graphs.
- **DAGCN**. DAGCN adopts dual attention structure, which can extract information from different hop. The network uses an innovative self‐attentional pool technique to represent graph information as an embedded matrix, which maximizes the raw information behind the graph. Since the attention mechanism is also used in our method, DAGCN represents an important contrast.
- **DGCN**. The model adopts two simple parallel feed‐forward networks local consistency convolution and global consistency convolution. The difference is only that the input graph structure information is different, and the convolution parameters of the two parallel graphs are shared. After the two branches, the loss function is added to combine local consistency and global consistency, which makes good use of the prior knowledge of the original data.
- **GREEN**. GREEN proposes the Graph REsidual rE‐ranking Network, which introduced class dependency module. Through GCN, the module can automatically learn the relationship between levels in the feature space, and combine this relationship as residual with the original classification result to obtain the final classification result.
- **HGCN**. This method adopts hybrid GCN, which combines CNN and GCN, and introduces a modularity‐based graph learning module. The GCN features of the graph are obtained via graph learning module, and the semi‐supervised classification method is used to optimize the classification performance.

### 3.4 Experimental results

We implement our network based on Pytorch. The optimizer of network training chooses stochastic gradient descent (SGD) optimizer,^37^ the momentum is 0.9 and the weight decay is 0.0001. The initial learning rate is set to 0.0001 and the learning rate decay strategy is divided by 10 for every 20 epochs and train 60 epochs with a batch size of 32. Hyperparameter α is set to 0.2. According to the above framework, 10‐fold cross‐validation is adopted to improve the generalization ability.

**FIGURE 5:**
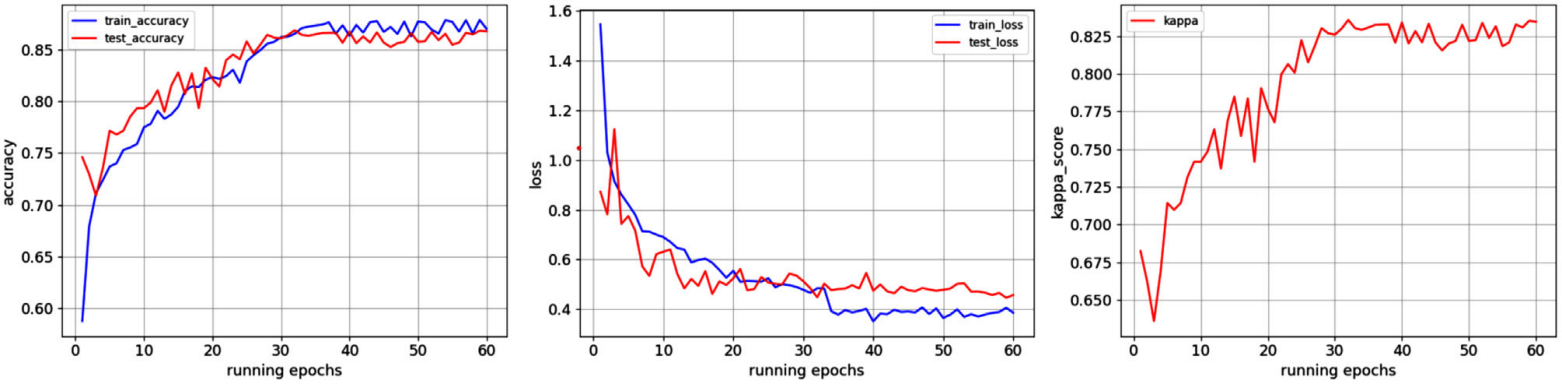
Illustration of graph attentional convolutional neural network on APTOS dataset. Our models are trained in 60 epochs. The figures are accuracy‐epoch, running‐epoch, kappa‐epoch curves in turn. The blue and red curves represent the training and testing processes, respectively

Figure 5 shows the training and testing result curves of the method in this paper on APTOS dataset. We train and test the model with 60 epochs. Accuracy and loss began to stabilize after the 32nd epoch.

We set the labeling rate to 1%, 2%, 5%, 10%, 20%, 25%, 50%, and 100% for testing. In Table 3, the classification accuracy of different labeling rates is given. Accuracy is relatively low when the labeling rate is 1% or 2%. With the increase of the labeling rates, the classification accuracy is higher. When the labeling rate is set to 100%, the model only uses labeled data for fully supervised training. At this time, the classification accuracy of graph attentional convolutional neural network (GACNN) reaches 93.35%, compared with DenseNet121, the accuracy is improved by 6.24%. Also, compared with DAGCN, the accuracy is improved by 4.9%.

**TABLE 3:** Comparison of our proposed method with other methods on APTOS dataset (%)

**Labeling rate (%)**	**Index**	**Dense Net121**	**Deep Walk**	**DAGCN**	**DGCN**	**GREEN**	**HGCN**	**GACNN**
1	Precision	63.65	64.34	64.65	65.10	65.79	66.21	65.74
	Recall	63.49	64.10	64.56	64.79	65.56	65.87	65.79
	Kappa	54.36	55.13	55.70	55.99	56.95	57.34	57.24
	Accuracy	63.49	64.10	64.56	64.79	65.56	65.87	65.79
2	Precision	72.87	74.12	73.57	72.99	75.31	73.21	77.09
	Recall	72.35	73.87	73.56	72.98	75.12	73.12	76.98
	Kappa	65.44	67.34	66.95	66.23	68.90	66.40	71.23
	Accuracy	72.35	73.87	73.56	72.98	75.12	73.12	76.98
5	Precision	78.86	80.12	80.36	80.32	83.35	82.18	83.33
	Recall	78.86	80.05	80.26	80.23	83.11	82.15	83.27
	Kappa	73.58	75.06	75.33	75.29	78.89	77.69	79.09
	Accuracy	78.86	80.05	80.26	80.23	83.11	82.15	83.27
10	Precision	80.57	81.36	82.67	82.92	84.32	84.87	85.66
	Recall	80.53	81.14	82.57	82.90	84.31	84.82	85.59
	Kappa	75.66	76.43	78.21	78.63	80.39	81.03	81.99
	Accuracy	80.53	81.14	82.57	82.90	84.31	84.82	85.59
20	Precision	80.68	81.54	82.79	83.1	86.67	87.01	86.68
	Recall	80.97	81.36	82.77	82.96	86.44	86.98	86.54
	Kappa	76.21	76.70	78.46	78.70	83.05	83.73	83.18
	Accuracy	80.97	81.36	82.77	82.96	86.44	86.98	86.54
25	Precision	81.69	81.55	83.07	83.34	88.06	88.13	87.87
	Recall	80.98	81.43	82.98	83.24	87.58	88.12	87.86
	Kappa	76.23	76.79	78.73	79.05	84.48	85.15	84.83
	Accuracy	80.98	81.43	82.98	83.24	87.58	88.12	87.86
50%	Precision	83.68	83.69	85.01	84.99	90.25	90.15	89.83
	Recall	83.45	83.75	84.78	84.97	90.26	90.07	89.79
	Kappa	79.31	79.69	80.98	81.21	87.83	87.59	87.24
	Accuracy	83.45	83.75	84.78	84.97	90.26	90.07	89.79
**100**	Precision	87.13	87.22	88.63	88.54	93.89	93.96	**93.37**
	Recall	87.11	87.34	88.45	88.54	93.68	93.79	**93.35**
	Kappa	83.89	84.18	85.56	85.68	92.10	92.24	**91.69**
	Accuracy	87.11	87.34	88.45	88.54	93.68	93.79	**93.35**

**FIGURE 6:**
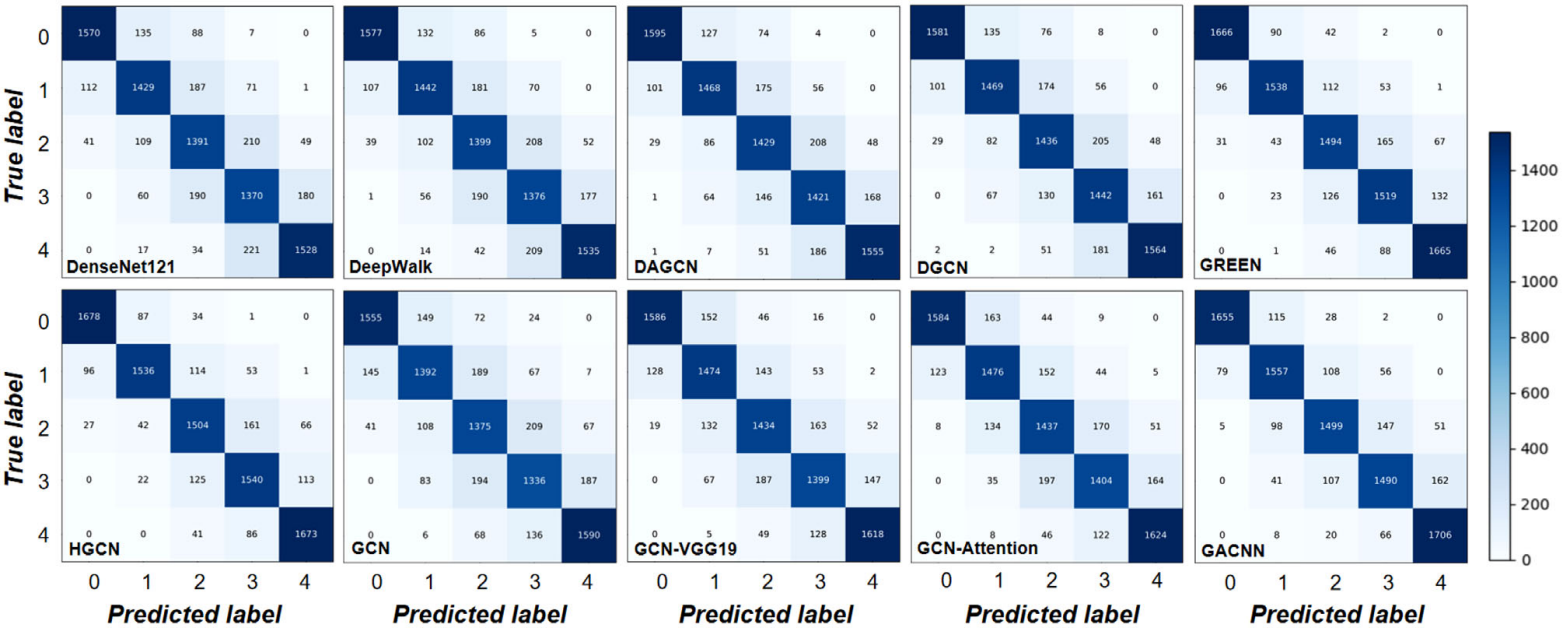
Confusion matrix of multiple classification networks. The abscissa represents the predicted label and the ordinate represents the true label. Diagonal elements represent samples with correct classification. The darker the color, the higher the sample value and classification accuracy

In addition, we also use other visualization results. Figure 6 shows the confusion matrixes of these models. The colors on the diagonal represent the performance of the classification. In Figure 6, it can be clearly seen that the diagonal colors of DenseNet121, DeepWalk, and GCN are lighter. Compared with other categories, the classification error rates of the two categories of images labeled ‘2’ and ‘3’ are relatively high.

**FIGURE 7:**
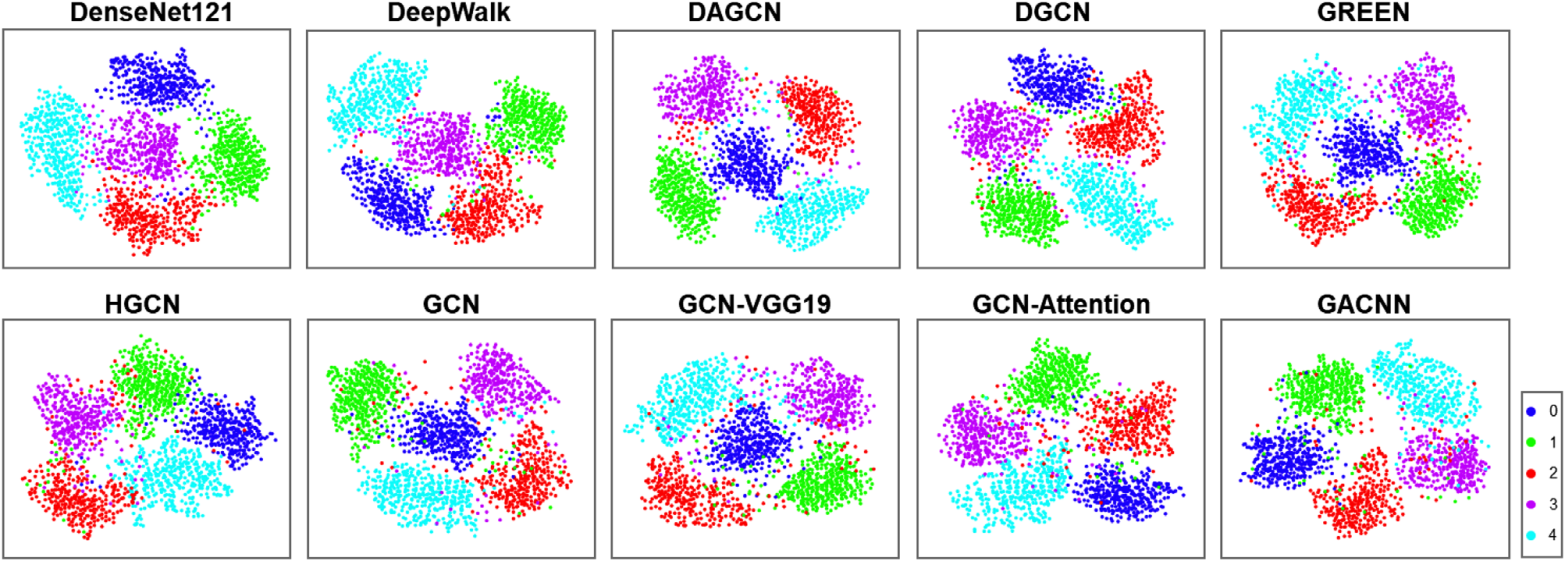
2D t‐distributed stochastic neighbor embedding visualizations of the feature map output on APTOS dataset. Five different colors represent five categories of images

Figure 7 shows the t‐distributed stochastic neighbor embedding (t‐SNE)^38^ visualization of the feature maps output by each network. We use five colors to mark the five categories. We can find that after embedding high‐dimensional data into two‐dimensional space through t‐SNE, the category information between data is retained, and the distribution between different categories is obvious.

### 3.5 Influence of semi‐supervised method on model performance

To verify the effectiveness of semi‐supervised learning, we compared the two methods of using only labeled data and both labeled and unlabeled data. We compared the experimental results with the accuracy of the method that simultaneously used labeled data and unlabeled data in Table 3, as shown in Table 4. We set the labeling rate to 1%, 2%, 5%, 10%, 20%, 25%, and 50%, and input only labeled data (no unlabeled data) to our model.

**TABLE 4:** Comparison of graph attentional convolutional neural network with/without semi‐supervised learning (%)

		**Whether unlabeled data is used**	
**Architecture**	**Labeling rate (%)**	×	√
GACNN	1	60.52	65.79
	2	72.39	76.98
	5	79.84	83.27
	10	83.64	85.59
	20	85.99	86.54
	25	87.46	87.86
	50	89.48	89.79

It can be seen from Table 4 that the model using semi‐supervised learning method achieves higher accuracy. For example, the accuracy of the proposed method with 1% labeled data and 99% unlabeled data is 65.79%, which is higher than that of 1% labeled data.

### 3.6 Effect of regularization weight λ

**FIGURE 8:**
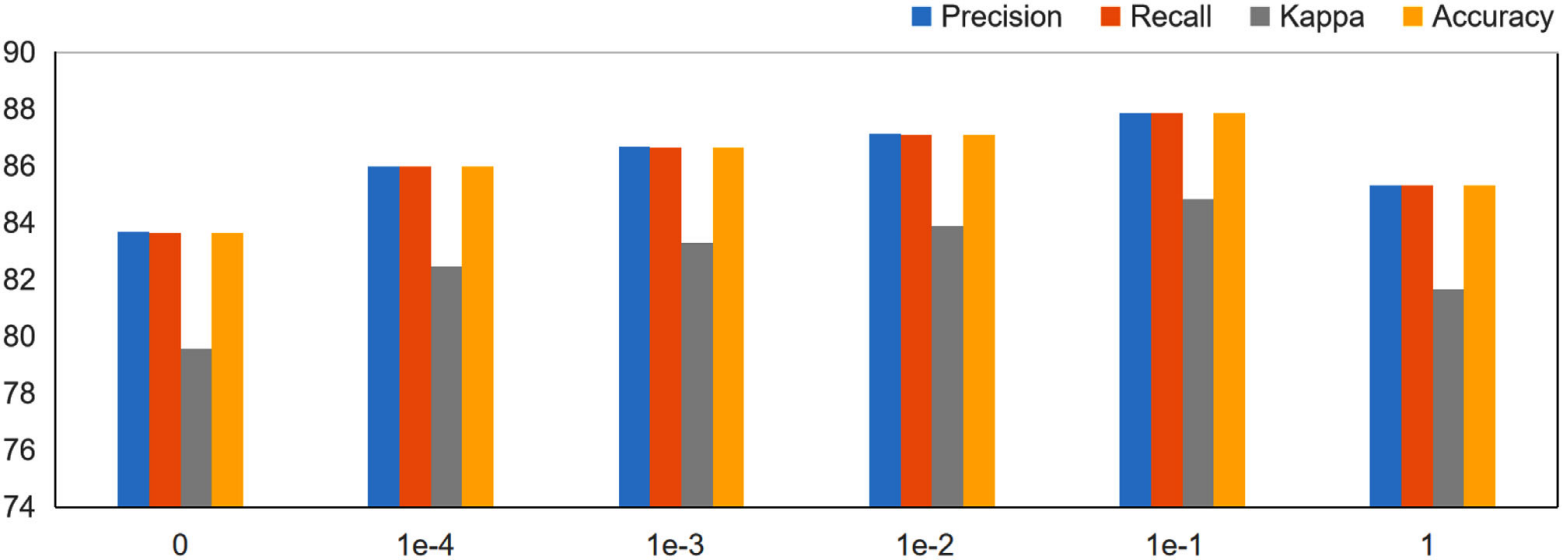
Results of graph attentional convolutional neural network with different settings of parameter λ

In formula (9), GACNN uses hyperparameters λ to balance two loss functions. In the following experiments, we evaluate the performance of GACNN with different hyperparametric settings. Figure 8 shows the experimental results under different parameter settings. Classification performance is the best when λ is set to 1e‐1 or 1e‐2.

### 3.7 Ablation study

In order to further verify the effectiveness of the network, we conducted ablation experiments. We conducted experiments on the network without attention mechanism and the GCN based on attention mechanism. Table 5 compares the impact of each part on the performance of network classification, and analyzes the effectiveness of CNN branch and attention mechanism on the model. Compared with GCN, the accuracy of our method is improved by 5.71%, which is due to the combination of the global feature extracted by CNN and the spatial feature extracted by GCN. In addition, the introduction of attention mechanism improves the adaptability of GCN to topology and increases the weight of target nodes. In general, the proposed method can effectively improve the classification accuracy and enhance the generalization ability of the model.

**TABLE 5:** Comparison results between graph attentional convolutional neural network (GACNN) and variants of GACNN when training size is 25% (%)

**Methods**	**Precision**	**Recall**	**Kappa**	**Accuracy**
GCN	82.16	82.15	77.69	82.15
VGG‐19	83.49	83.51	79.39	83.51
GCN‐VGG19	84.91	84.83	81.04	84.83
GCN‐Attention	85.35	85.27	81.59	85.27
**GACNN**	**87.87**	**87.86**	**84.83**	**87.86**

## 4 DISCUSSION

In this paper, we proposed a classification model of lesion degree of fundus images—GACNN. The main advantages of our proposed method are as follows: on the one hand, GACNN combines CNN and GCN to extract local and global features of fundus images, and uses attention mechanism to aggregate the graph information. On the other hand, we expanded the train set before training process, which can improve the robustness of the model. In addition, the model improves the generalization ability of the network by combining two loss functions to guide the learning process. However, Table 3 shows that when the marking rate is a specific value, the classification results of our method are inferior to those of GREEN and HGCN, which may be due to the difference between class dependency module and graph learning module. In GREEN, the author inputs the image classes as graph nodes into GCN, and multiplies the output class adjacency matrix with the feature vector output by CNN to update the weight, and output the predicted value. GREEN directly processes category information rather than image features. In HGCN, the idea of graph learning module is to input the image features extracted by CNN into GCN, use GCN to build the adjacency matrix of nodes, and finally use two‐part loss function for learning.

The performance of the model is verified by the experiments in the previous section. Table 3 compares the experimental results of various models under different labeling rates. With the increase of labeling rate, the performance of the model is gradually improved. When the labeling rate is set to 100%, the classification accuracy, precision, and recall reach the highest in the experiment. Except for GREEN and HGCN, GACNN performs best in all other models.

Since labeled data requires a huge workload of annotation, semi‐supervised learning is efficient to train a comparable model, which can also take advantage of the unlabeled data. Table 4 demonstrate the results of GACNN with/without semi‐supervised learning, it shows that the unlabeled data can help improve the accuracy of the model with only the labeled data, and we can conclude that semi‐supervised learning method has the potential of improving the classification performance.

Particularly, our method uses the similar attention mechanism strategy as DAGCN, but shows better performance on this dataset, which shows that the combination of VGG and GCN is effective for improving classification performance. In Table 5, we compared the classification results of GACNN and its variants on the APTOS dataset. The results show that GACNN has the best accuracy. GCN can automatically learn node characteristics and association information between nodes. We construct the graph structure to obtain the relationship between different lesion regions, so that the network can learn the differences between the lesion features of various images, so as to better classify the images. The accuracy of our model is significantly higher than that of the model without attention mechanism, which indicates the influence of attention mechanism in the model. Attention mechanism helps the network identify local lesion areas, enhances the region of interest, and reduces the recognition of irrelevant areas.

**FIGURE 9:**
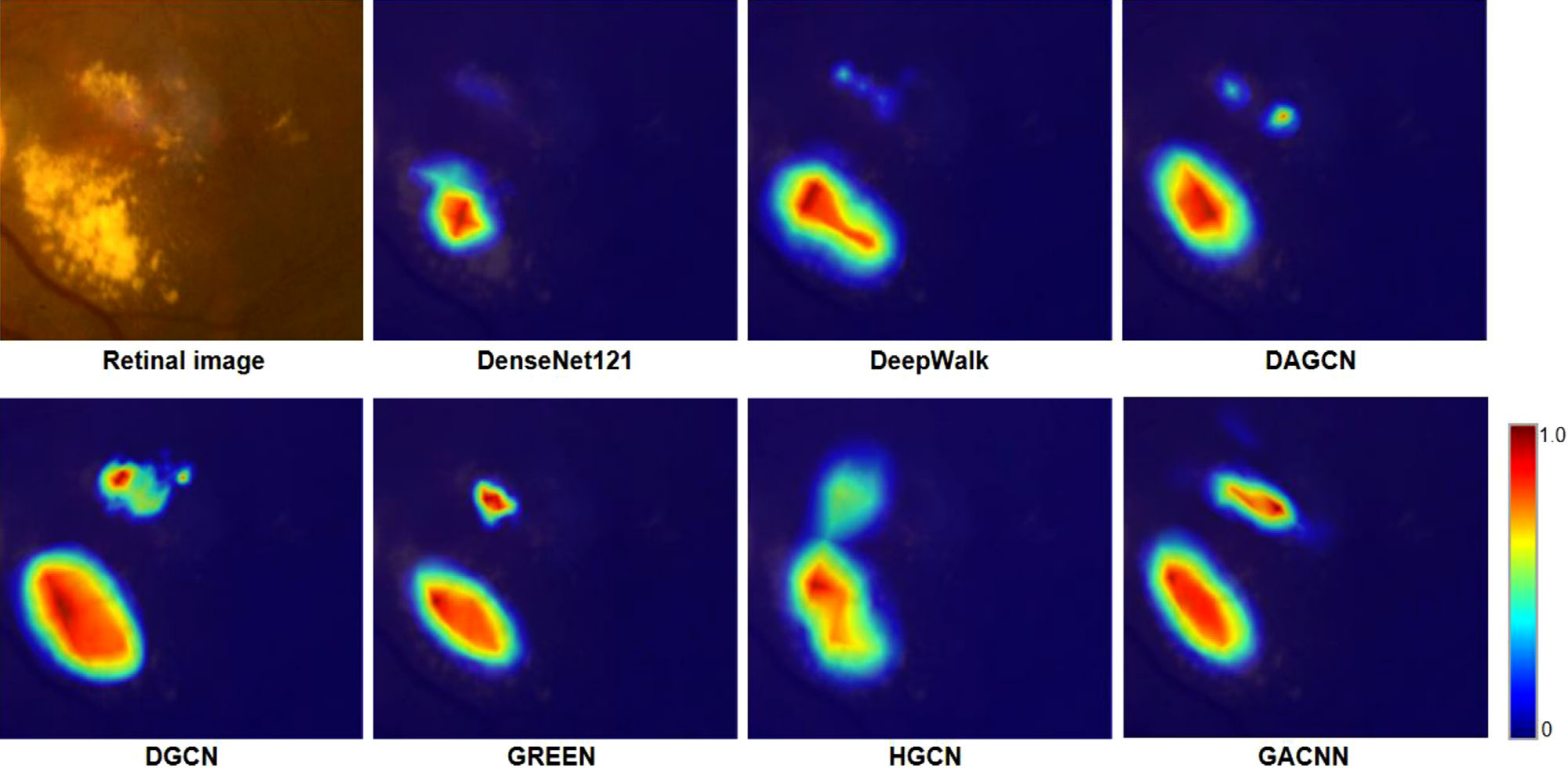
Visual explanation of each model. Red represents higher weight, while blue represents lower weight

We visualize the features learned by the model and highlight the important areas in the image for classification to show the decision‐making mode of classification. Figure 9 shows the visualization results of Gradient‐weighted Class Activation Map (Grad‐CAM) of each model.

Figures 6 and 7 show the visual experimental results of each model. The performance of our method is better than other baselines. It can be seen from the figures that DenseNet121 and DeepWalk do not have clear classification boundaries for different classes of images. Due to the complexity of fundus images and the diversity and similarity of lesion types, it is difficult for some networks to identify DR images with similar lesion types. Therefore, our model ameliorates this problem.

However, the classification performance of the model for the categories labeled 2 and 3 is poor, which may be due to the similarity of the two classes of image features. Images labeled 2 are mostly represented by the appearance of white spots, while images labeled 3 are mostly represented by the appearance of cotton flocculent leukoplakia in the fundus. However, white spots and bleeding spots often exist in images labeled 3, which leads to the similarity between the two classes of images.

Finally, we study the influence of different hyperparameters λ on network performance. As shown in Figure 8, when λ is set to 0, the experimental results decrease significantly, and the loss function only depends on the previous item, which is also feasible in the experiment.

However, our experiment still has some limitations. The amount of data in our experiment is insufficient and the category distribution is uneven. Our study only considers the images after data enhancement. In the future, we intend to implement our model on more datasets. We will also take a deeper look at the graph convolution and study whether our model can be applied to other research fields.

## 5 CONCLUSION

In this work, we propose a semi‐supervised classification model based on CNN and GCN. In addition, we introduce attention mechanism into the GCN branch to reduce the information loss in the traditional graph convolution step. We compared GACNN with other network models, and the results show that the proposed method can significantly improve the classification performance of DR images. Finally, we conducted ablation experiments to verify the attention mechanism and the effectiveness of feature fusion between the two networks. The combination of the two networks and the attention mechanism can effectively enhance the ability of extracting the hierarchical information of image.

## AUTHOR CONTRIBUTIONS

*Sixu Duan: Conceived the idea of the study, designed and conducted the experiments, performed the analysis, and wrote the paper*. Dengwang Li: Supervision. *Min Chen: Guidance of clinical knowledge*. Ting Wang: *Visualization and investigation*. Xiaolei Sun: *Software and validation*. Meirong Chen: *Investigation*. Xueyuan Dong: *Data curation*. Zekun Jiang: Validation. Pu Huang: *Writing, review and editing*. All authors contributed to revising the paper.

## CONFLICT OF INTEREST

The authors declare that they have no known competing financial interests or personal relationships that could have appeared to influence the work reported in this paper.
